# Overview: How Is Alcohol Metabolized by the Body?

**Published:** 2006

**Authors:** Samir Zakhari

**Affiliations:** Samir Zakhari, Ph.D., is director, Division of Metabolism and Health Effects, National Institute on Alcohol Abuse and Alcoholism, Bethesda, Maryland

**Keywords:** Ethanol-toacetaldehyde metabolism, alcohol dehydrogenase (ADH), aldehyde dehydrogenase (ALDH), acetaldehyde, acetate, cytochrome P450 2E1 (CYP2E1), catalase, reactive oxygen species (ROS), blood alcohol concentration (BAC), liver, stomach, brain, fetal alcohol effects, genetics and heredity, ethnic group, hypoxia

## Abstract

Alcohol is eliminated from the body by various metabolic mechanisms. The primary enzymes involved are aldehyde dehydrogenase (ALDH), alcohol dehydrogenase (ADH), cytochrome P450 (CYP2E1), and catalase. Variations in the genes for these enzymes have been found to influence alcohol consumption, alcohol-related tissue damage, and alcohol dependence. The consequences of alcohol metabolism include oxygen deficits (i.e., hypoxia) in the liver; interaction between alcohol metabolism byproducts and other cell components, resulting in the formation of harmful compounds (i.e., adducts); formation of highly reactive oxygen-containing molecules (i.e., reactive oxygen species [ROS]) that can damage other cell components; changes in the ratio of NADH to NAD^+^ (i.e., the cell’s redox state); tissue damage; fetal damage; impairment of other metabolic processes; cancer; and medication interactions. Several issues related to alcohol metabolism require further research.

The effects of alcohol (i.e., ethanol) on various tissues depend on its concentration in the blood (blood alcohol concentration [BAC]) over time. BAC is determined by how quickly alcohol is absorbed, distributed, metabolized, and excreted. After alcohol is swallowed, it is absorbed primarily from the small intestine into the veins that collect blood from the stomach and bowels and from the portal vein, which leads to the liver. From there it is carried to the liver, where it is exposed to enzymes and metabolized. The rate of the rise of BAC is influenced by how quickly alcohol is emptied from the stomach and the extent of metabolism during this first pass through the stomach and liver (i.e., first-pass metabolism [FPM]).

BAC is influenced by environmental factors (such as the rate of alcohol drinking, the presence of food in the stomach, and the type of alcoholic beverage) and genetic factors (variations in the principal alcohol-metabolizing enzymes alcohol dehydrogenase [ADH] and aldehyde dehydrogenase [ALDH2]). The alcohol elimination rate varies widely (i.e., three-fold) among individuals and is influenced by factors such as chronic alcohol consumption, diet, age, smoking, and time of day (Bennion and Li 1976; Kopun and Propping 1977).

The consequent deleterious effects caused by equivalent amounts of alcohol also vary among individuals. Even after moderate alcohol consumption, BAC can be considerable (0.046 to 0.092 gram-percent [g%]; in the 10- to 20-millimolar^1^ [mM] range). Alcoholreadily diffuses across membranes and distributes through all cells and tissues, and at these concentrations, it can acutelyaffect cell function by interacting with certain proteins and cell membranes. As explained in this article, alcohol metabolism also results in the generation of acetaldehyde, a highly reactive and toxic byproduct that may contribute to tissue damage, the formation ofdamaging molecules known as reactive oxygen species (ROS), and a change in the reduction–oxidation (or redox) state of liver cells. Chronic alcohol consumption and alcohol metabolism are strongly linked to several pathological consequences and tissue damage.

Understanding the balance of alcohol’s removal and the accumulation of potentially damaging metabolic byproducts, as well as how alcohol metabolism affects other metabolic pathways, is essentialfor appreciating both the short-term and long-term effects of the body’s response to alcohol intake.

## Alcohol Metabolism

Although the liver is the main organ responsible for metabolizing ingested alcohol, stomach (i.e., gastric) ADH has been reported to contribute to FPM. The relative contribution of the stom ach and the liver to FPM, however, is controversial. Thus, whereas FPM is attributed predominantly to the stomach (Lim et al. 1993; Baraona 2000), other previous studies (Lee et al. 2006) stress the role of the liver. Human ADH3, which is present in the liver and stomach, metabolizes alcohol poorly at physiological BACs (i.e., 0.23 g% BAC [or <50 mM]) in the liver but may play an important role in FPM in the stomach, because gastric alcohol concentrations can reach molar range during alcohol consumption (Baraona et al. 2001; Lee et al. 2003). However, Crabb (1997) pointed out the insufficiency of gastric ADH to account for FPM, so this remains unresolved.

Alcohol also is metabolized in nonliver (i.e., extrahepatic) tissues that do not contain ADH, such as the brain, by the enzymes cytochrome P450 and catalase (see below). In general, alcohol metabolism is achieved by both oxidative pathways, which either add oxygen or remove hydrogen (through pathways involving ADH, cytochrome P450, and catalase enzymes), and nonoxidative pathways.

## Oxidative Pathways

**Figure 1:**
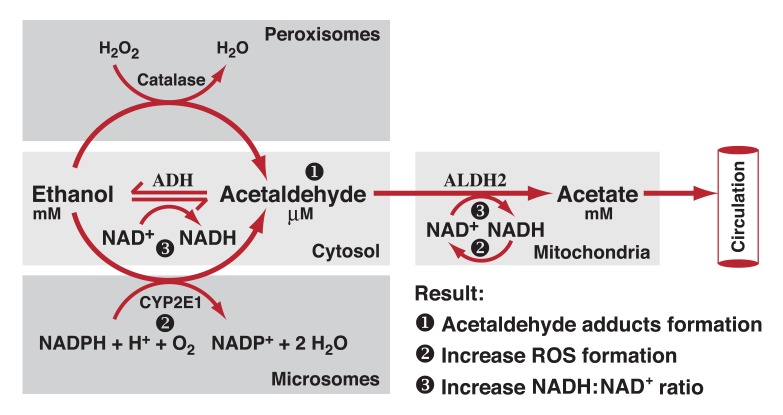
Oxidative pathways of alcohol metabolism. The enzymes alcohol dehydrogenase (ADH), cytochrome P450 2E1 (CYP2E1), and catalase all contribute to oxidative metabolism of alcohol. ADH, present in the fluid of the cell (i.e., cytosol), converts alcohol (i.e., ethanol) to acetaldehyde. This reaction involves an intermediate carrier of electrons, ^+^nicotinamide adenine dinucleotide (NAD), which is reduced by two electrons to form NADH. Catalase, located in cell bodies called peroxisomes, requires hydrogen peroxide (H_2_O_2_) to oxidize alcohol. CYP2E1, present predominantly in the cell’s microsomes, assumes an important role in metabolizing ethanol to acetaldehyde at elevated ethanol concentrations. Acetaldehyde is metabolized mainly by aldehyde dehydrogenase 2 (ALDH2) in the mitochondria to form acetate and NADH. ROS, reactive oxygen species.

As shown in Figure 1, ADH, cytochrome P450 2E1 (CYP2E1), and catalase all contribute to oxidative metabolism of ethanol.

### ADH

The major pathway of oxidative metabolism of ethanol in the liver involves ADH (present in the fluid of the cell [i.e., cytosol]), an enzyme with many different variants (i.e., isozymes). Metabolism of ethanol with ADH produces acetaldehyde, a highly reactive and toxic byproduct that may contribute to tissue damage and, possibly, the addictive process. As shown in Table 1, ADH constitutes a complex enzyme family, and, in humans, five classes have been categorized based on their kinetic and structural properties. At high concentrations, alcohol is eliminated at a high rate because of the presence of enzyme systems with high activity levels (*K*_m_),^2^ such as class II ADH, β_3_-ADH (encoded by *ADH4* and *ADH1B* genes, respectively) and CYP2E1 (Bosron et al. 1993). This oxidation process involves an intermediate carrier of electrons, nicotinamide adenine dinucleotide (NAD^+^), which is reduced by two electrons to form NADH. As a result, alcohol oxidation generates a highly reduced cytosolic environment in liver cells (i.e., hepatocytes). In other words, these reactions leave the liver cells in a state that is particularly vulnerable to damage from the byproducts of ethanol metabolism, such as free radicals and acetaldehyde.

### Cytochrome P450

The cytochrome P450 isozymes, including CYP2E1, 1A2, and 3A4, which are present predominantly in the microsomes, or vesicles, of a network of membranes within the cell known as the endoplasmic reticulum, also contribute to alcohol oxidation in the liver. CYP2E1 is induced by chronic alcohol consumption and assumes an important role in metabolizing ethanol to acetaldehyde at elevated ethanol concentrations (*K*_m_ = 8 to 10 mM, compared with 0.2 to 2.0 mM for hepatic ADH). In addition, CYP2E1dependent ethanol oxidation may occur in other tissues, such as the brain, where ADH activity is low. It also produces ROS, including hydroxyethyl, superoxide anion, and hydroxyl radicals, which increase the risk of tissue damage.

### Catalase

Another enzyme, catalase, located in cell bodies called peroxisomes, is capable of oxidizing ethanol in vitro in the presence of a hydrogen peroxide (H_2_O_2_)-generating system, such as the enzyme complex NADPH oxidase or the enzyme xanthine oxidase. Quantitatively, however, this is considered a minor pathway of alcohol oxidation, except in the fasted state (Handler and Thurman 1990). Chronic alcohol consumption by rats has been shown to result in increased H_2_O_2_ production in pericentral regions of the liver and increased catalase activity (Misra et al. 1992). The role of CYP2E1 and catalase in alcohol metabolism in the brain are described in detail elsewhere (Zimatkin and Deitrich 1997).

### Products of Oxidative Metabolism of Alcohol

Acetaldehyde and acetate, produced from the oxidative metabolism of alcohol, contribute to cell and tissue damage in various ways.

#### Acetaldehyde

Acetaldehyde, produced by alcohol oxidation through any of the mechanisms outlined above, is rapidly metabolized to acetate, mainly by ALDH2 (in cell bodies called mitochondria), to form acetate and NADH. NADH then is oxidized by a series of chemical reactions in the mitochondria (i.e., the mitochondrial electron transport chain, or respiratory chain). Acetaldehyde has the capacity to bind to proteins such as enzymes, microsomal proteins, and microtubules. It also forms adducts with the brain signaling chemical (i.e., neurotransmitter) dopamine to form salsolinol, which may contribute to alcohol dependence, and with DNA to form carcinogenic DNA adducts such as 1,N^2^-propanodeoxyguanosine. Formation of protein adducts in hepatocytes impairs protein secretion, which has been proposed to play a role in enlargement of the liver (i.e., hepatomegaly).

#### Acetate

Acetate, produced from the oxidation of acetaldehyde, is oxidized to carbon dioxide (CO_2_). Most of the acetate resulting from alcohol metabolism escapes the liver to the blood and is eventually metabolized to CO_2_ in heart, skeletal muscle, and brain cells. Acetate is not an inert product; it increases blood flow into the liver and depresses the central nervous system, as well as affects various metabolic processes (Israel et al. 1994). Acetate also is metabolized to acetyl CoA, which is involved in lipid and cholesterol biosynthesis in the mitochondria of peripheral and brain tissues. It is hypothesized that upon chronic alcohol intake the brain starts using acetate rather than glucose as a source of energy.

## Nonoxidative Pathways

**Figure 2:**
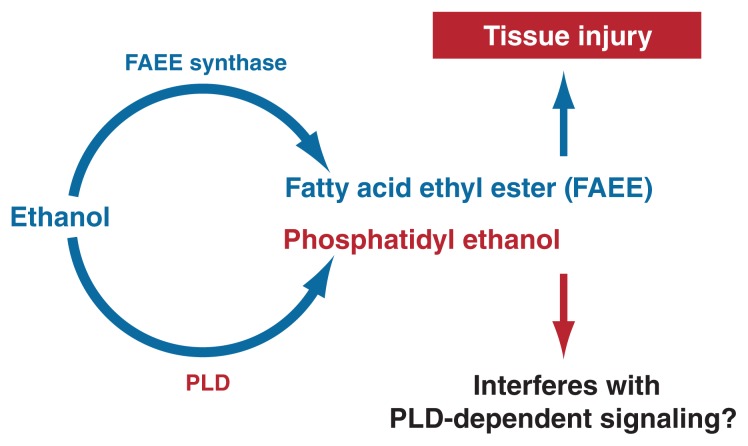
Ethanol is nonoxidatively metabolized by two pathways. A reaction catalyzed by the enzyme fatty acid ethyl ester (FAEE) synthase leads to the formation of molecules known as FAEEs. A reaction with the enzyme phospholipase D (PLD) results in the formation of a phospholipid known as phosphatidyl ethanol.

The nonoxidative metabolism of alcohol is minimal, but its products may have pathological and diagnostic relevance. Alcohol is nonoxidatively metabolized by at least two pathways. One leads to the formation of molecules called fatty acid ethyl esters (FAEEs) from the reaction of alcohol with fatty acids––weak organic acids that play functional roles in human cells. The other nonoxidative pathway results in the formation of a type of fat molecule (i.e., lipid) containing phosphorus (i.e, phospholipid) known as phosphatidyl ethanol (see Figure 2). FAEEs are detectable in serum and other tissues after alcohol ingestion and persist long after alcohol is eliminated. The role of FAEEs in alcohol-induced tissue damage remains to be further evaluated.

The second nonoxidative pathway requires the enzyme phospholipase D (PLD) (Laposata 1999), which breaks down phospholipids (primarily phosphatidylcholine) to generate phosphatidic acid (PA). This pathway is a critical component in cellular communication. PLD has a high *K*_m_ for ethanol, and the enzymatic reaction does occur predominantly at high circulating alcohol concentrations. The product of this reaction, phosphatidyl ethanol, is poorly metabolized and may accumulate to detectable levels following chronic consumption of large amounts of alcohol, but its effects on the cell remain to be established. However, the formation of phosphatidyl ethanol occurs at the expense of the normal function of PLD, namely to produce PA, resulting in inhibited PA formation and disruption of cell signaling.

Oxidative and nonoxidative pathways of alcohol metabolism are interrelated. Inhibition of ethanol oxidation by compounds that inhibit ADH, CYP2E1, and catalase results in an increase in the nonoxidative metabolism of alcohol and increased production of FAEEs in the liver and pancreas (Werner et al. 2002).

## Genetic Aspects of Alcohol Metabolism

Variations in the rate of alcohol absorption, distribution, and elimination contribute significantly to clinical conditions observed after chronic alcohol con sumption. These variations have been attributed to both genetic and environmental factors, gender, drinking pattern, fasting or fed states, and chronic alcohol consumption. The following section will focus on the relevant genetic factors.

### Genetic Variation in ADH and ALDH

Class I ADH and ALDH2 play a central role in alcohol metabolism. Variations in the genes encoding ADH and ALDH produce alcohol-and acetaldehyde-metabolizing enzymes that vary in activity. This genetic variability influences a person’s susceptibility to developing alcoholism and alcohol-related tissue damage.

#### ADH

The ADH gene family encodes enzymes that metabolize various substances, including ethanol. The activity of these enzymes varies across different organs (see Table 1). When ethanol is present, the metabolism of the other substances that ADH acts on may be inhibited, which may contribute to ethanol-induced tissue damage.

As shown in Table 1, genetic variation (i.e., polymorphism) occurs at the *ADH1B* and *ADH1C* gene locations (see Agarwal 2001), and these different genes are associated with varying levels of enzymatic activity. The *ADH1B* variations (i.e., alleles) occur at different frequencies in different populations. For example, the ADH1B*1 form is found predominantly in Caucasian and Black populations, whereas ADH1B*2 frequency is higher in Chinese and Japanese populations and in 25 percent of people with Jewish ancestry. ADH1C*1 and ADH1C*2 appear with roughly equal frequency in Caucasian populations (Li 2000). People of Jewish descent carrying the ADH1B*2 allele show only marginally (<15 percent) higher alcohol elimination rates compared with people with ADH1B*1 (Neumark et al. 2001). Also, African Americans (Thomasson et al. 1995) and Native Americans (Wall et al. 1996) with the ADH1B*3 allele metabolize alcohol at a faster rate than those with ADH1B*1.

#### ALDH

Several isozymes of ALDH have been identified, but only the cytosolic ALDH1 and the mitochondrial ALDH2 metabolize acetaldehyde. There is one significant genetic polymorphism of the *ALDH2* gene, resulting in allelic variants ALDH2*1 and ALDH2*2, which is virtually inactive. ALDH2*2 is present in about 50 percent of the Taiwanese, Han Chinese, and Japanese populations (Shen et al. 1997) and shows virtually no acetaldehyde metabolizing activity in vitro. People who have one (i.e., heterozygous) or especially two (i.e., homozygous) copies of the ALDH2*2 allele show increased acetaldehyde levels after alcohol consumption (Luu et al. 1995; Wall et al. 1997) and therefore experience negative physiological responses to alcohol.

Because polymorphisms of ADH and ALDH2 play an important role in determining peak blood acetaldehyde levels and voluntary ethanol consumption (Quintanilla et al. 2005), they also influence vulnerability to alcohol dependence. A fast ADH or a slow ALDH are expected to elevate acetaldehyde levels and thus reduce alcohol drinking. These polymorphisms and their significance are discussed in the article in this issue by Quertemont and Didone.

ADH and ALDH isozyme activity also influences the prevalence of alcohol-induced tissue damage. Alcoholic cirrhosis is reduced more than 70 percent in populations carrying the ALDH2*2 allele (Chao et al. 1994; Nagata et al. 2002). In a review of studies, Yokoyama and Omori (2003) reported a positive correlation between genetic polymorphisms for low-activity ADH and ALDH and esophageal and head and neck cancers. In another study (Hines et al. 2001), moderate drinkers who are homozygous for the slow-oxidizing ADH1C*2 allele, and therefore who are expected to drink at higher levels than those with the ADH1C*1 allele, showed a substantially decreased risk of heart attack (i.e., myocardial infarction). The authors (Hines et al. 2001, p. 549) only differentiated drinkers versus nondrinkers at one drink per day (“Men who consumed at least one drink per day and were homozygous for the gamma2 allele had the greatest reduction in risk [relative risk 0.14]”).

Interestingly, elevated acetaldehyde levels induced by ALDH inhibitors were shown to protect against alcohol-induced liver injury in experimental animals (Lindros et al. 1999) and to reduce the release of a signaling molecule (i.e., cytokine) called tumor necrosis factor alpha (TNF-α) from Kupffer cells (Nakamura et al. 2004). This finding is quite contradictory to the belief that acetaldehyde plays a role in liver damage. In a meta-analysis of most studies in the literature, Zintzaras and colleagues (2005) found that neither ADH nor ALDH alleles were significantly associated with liver cirrhosis.

### Genetic Variation in CYP2E1

Although several *CYP2E1* polymorphisms have been identified, only a few studies were undertaken to determine the effect on alcohol metabolism and tissue damage. In one study (Ueno et al. 1996), the presence of the rare c2 allele was associated with higher alcohol metabolism in Japanese alcoholics but only at high BACs (0.25 g/dL). Raimondi and colleagues (2004) reported that study participants with a polymorphism of CYP2E1 (CYP2E1 RsaI) were more likely than others to be lifetime abstainers at age 68 or older. Burim and colleagues (2004) found an association between having the m2/m2 *CYP1A1* gene and alcoholic liver cirrhosis and the Val/Val *GSTP1* (glutathione *S*-transferase) gene and chronic pancreatitis.

## Consequences of Alcohol Metabolism

The different pathways of ethanol metabolism described above have numerous detrimental consequences that contribute to the tissue damage and diseases seen in alcoholic patients. These consequences include oxygen deficits (i.e., hypoxia) in the liver; interaction between alcohol metabolism byproducts and other cell components, resulting in the formation of harmful compounds (i.e., adducts); formation of highly reactive oxygen-containing molecules (i.e., reactive oxygen species [ROS]) that can damage other cell components; and changes in the ratio of NADH to NAD^+^ (i.e., the cell’s redox state [see Glossary]). These consequences and the way they contribute to tissue damage and disease will be discussed in the following sections.

### Hypoxia

As mentioned earlier, the main pathway of alcohol metabolism, which involves ADH and ALDH, results in the generation of NADH. The NADH then is oxidized by a series of chemical reactions in the mitochondria (i.e., the mitochondrial electron transport system, or respiratory chain), eventually resulting in the transfer of electrons to molecular oxygen (O_2_), which then binds protons (H^+^) to generate water (H_2_O). To have enough oxygen available to accept the electrons, the hepatocytes must take up more oxygen than normal from the blood. Consistent with this assumption, studies have shown that ethanol metabolism tends to increase the hepatocytes’ oxygen uptake from the blood (Tsukamoto and Xi 1989). If the hepatocytes that are located close to the artery supplying oxygen-rich blood to the liver take up more than their normal share of oxygen, however, not enough oxygen may be left in the blood to adequately supply other liver regions with oxygen. Indeed, strong evidence suggests that alcohol consumption results in significant hypoxia in those hepatocytes that are located close to the vein where the cleansed blood exits the liver (i.e., in the perivenous hepatocytes) (Arteel et al. 1996). The perivenous hepatocytes also are the first ones to show evidence of damage from chronic alcohol consumption (Ishak et al. 1991), indicating the potential harmful consequences of hypoxia induced by ethanol metabolism.

In addition to directly increasing hepatocytes’ oxygen use as described above, ethanol indirectly increases the cells’ oxygen use by activating Kupffer cells in the liver. When these cells become activated, they release various stimulatory molecules. One of these molecules is prostaglandin E2, which stimulates the metabolic activity of hepatocytes—that is, it induces them to break down and synthesize many essential molecules through a variety of chemical reactions that also require oxygen. As a result, alcohol-induced Kupffer cell activation also contributes to the onset of hypoxia.

### Adduct Formation

Ethanol metabolism by ADH and CYP2E1 produce reactive molecules, such as acetaldehyde and ROS, that can interact with protein building blocks (i.e., amino acids) and other molecules in the cell to form both stable and unstable adducts (see Table 2).

#### Acetaldehyde Adducts

Acetaldehyde interacts with certain amino acids in proteins (e.g., lysine, cysteine, and some of a group of amino acids called aromatic amino acids). However, not all amino acids in all proteins are equally likely to interact with acetaldehyde, and certain proteins seem to be particularly susceptible to forming adducts with acetaldehyde. These include the following (Tuma and Casey 2003):

- Proteins found in the membranes surrounding the red blood cells (i.e., erythrocytes).
- Lipoproteins that consist of a protein and a fat component and which are associated with the risk of heart disease.
- Tubulin, a protein found in cell structures called microtubules that are essential for cell division and protein transport within cells.
- Hemoglobin, which is crucial for oxygen transport by the erythrocytes.
- Albumin, which is a protein found in the blood.
- Collagen, the major protein in connective tissue.
- Cytochrome enzymes, such as CYP2E1, which play a role in the metabolism of ethanol and many other substances.

Acetaldehyde–lysine adducts were detected in the plasma membrane of hepatocytes from alcohol-fed rats (Barry et al. 1987). These adducts can indirectly contribute to liver damage because the body recognizes them as “foreign” and therefore generates immune molecules (i.e., antibodies) against them. The presence of such antibodies has been demonstrated following chronic alcohol consumption (Israel et al. 1986). The antibodies bind to the adducts and induce the immune system to destroy the hepatocytes containing these adducts. This process is known as immune-mediated hepatotoxicity or antibody-dependent cell-mediated cytotoxicity (ADCC). Antibodies directed against other acetaldehyde–protein adducts also have been found in the blood of alcoholics (Lin et al. 1990; Worrall et al. 1990).

Adducts formed by the interaction of acetaldehyde with erythrocyte membranes have been detected in the erythrocytes of alcohol abusers. These adducts may be associated with ethanol-induced macrocytosis, a condition characterized by unusually large numbers of enlarged erythrocytes in the blood (Niemela and Parkkila 2004). Macrocytosis is a marker for alcohol abuse.

Finally, acetaldehyde can form adducts by interacting with compounds known as biogenic amines,^3^ which include, among others, neurotransmitters such as serotonin and dopamine. These adducts may have pharmacological effects on the nervous system. (For more information on adduct formation and other detrimental effects of acetaldehyde, see the article by Quertemont and Didone, p. 258).

#### ROS Formation

As mentioned earlier, ethanol metabolism by CYP2E1 and NADH oxidation by the electron transport chain generate ROS that results in lipid peroxidation. This process results in the formation of compounds known as malondialdehyde (MDA) and 4hydroxy-2-nonenal (HNE), both of which can form adducts with proteins (Worrall and Thiele 2001). In addition, acetaldehyde and MDA together can react with proteins to generate a stable MDA–acetaldehyde–protein adduct (MAA) (Tuma et al. 1996; Tuma 2002). All of these adducts can induce immune responses (e.g., the formation of antibodies) (Tuma and Casey 2003). Moreover, MAA adducts can induce inflammatory processes in certain types of liver cells (i.e., stellate cells and endothelial cells) (Tuma 2002). These and other findings indicate a link between MDA, HNE, and MAA adducts and subsequent development of liver disease (Tuma and Casey 2003).

### Formation of ROS and Decrease in Antioxidants

ROS, including superoxide (O_2_^•−^), hydrogen peroxide (H_2_O_2_), hypochlorite ion (OCl^−^), and hydroxyl (^•^OH) radicals, are naturally generated by many reactions in multiple regions of the cell. ROS act by “stealing” hydrogen atoms from other molecules, thereby converting those molecules into highly reactive free radicals. Alternatively, ROS can combine with stable molecules to form free radicals. Through both of these mechanisms, ROS play an important role in cancer development (i.e., carcinogenesis), atherosclerosis, diabetes, inflammation, aging, and other harmful processes. To prevent the damage these highly reactive compounds can cause, numerous defense systems have evolved in the body involving compounds called antioxidants, which can interact with ROS and convert them into harmless molecules. Under normal conditions, a balance between ROS and antioxidants exists in the cells. When this balance is disturbed and an excess of ROS is present, a state known as oxidative stress results.

In most cells, the vast majority of ROS are generated in association with the mitochondrial electron transport system. In addition, ROS are produced by CYP2E1 and by activated Kupffer cells in the liver. Both acute and chronic alcohol consumption can increase ROS production and lead to oxidative stress though a variety of pathways, including, but not limited to, the following (Wu and Cederbaum 2003):

- Changes in the cells’ redox state (i.e., in the ratio of NADH to NAD^+^) as a result of ethanol metabolism by ADH and ALDH, which results in production of more NADH.
- Acetaldehyde formation.
- Induction of CYP2E1 at high alcohol levels.
- Hypoxia related to alcohol metabolism.
- Alcohol-induced damage to the mitochondria.
- Activation of Kupffer cells.
- Reduction of the levels of certain antioxidants (e.g., mitochondrial and cytosolic glutathione).
- Formation of the 1-hydroxyethyl radical mentioned above.

The relative contributions of these factors to the increase in ROS levels is unknown (Bailey 2003). Regardless of how they were generated, however, increases in ROS levels have numerous detrimental effects. For example, ROS stimulate the release of TNF-α from Kupffer cells. This cytokine plays an important role in activating inflammatory reactions that can contribute to tissue damage and scar tissue formation (i.e., fibrosis) in the liver. In addition, ROS can interact with lipids, proteins, and DNA in a process called peroxidation, which can have harmful consequences. For example, as described in the previous section, lipid peroxidation leads to the generation of MDA and HNE. Peroxidation of mitochondrial membranes alters the membranes’ properties (e.g., membrane permeability) so that certain molecules that normally are contained in the mitochondria can escape the mitochondria into the cytosol. The release of a compound called cytochrome c into the cytosol, for example, induces a chain of biochemical reactions that ultimately causes a certain type of cell death (i.e., cell suicide, or apoptosis). Moreover, peroxidation of molecules in the mitochondrial membranes alters the distribution of electrical charges across the membrane, which results in reduced levels of ATP in the cell and promotes another type of cell death called necrosis. Both apoptosis and necrosis contribute to alcohol-related liver damage.

To prevent or ameliorate the harmful effects caused by ROS, researchers have studied the effects of antioxidant administration. These studies found that replenishment of glutathione by administering the glutathione precursor *S*-adenosyl-l-methionine (SAMe) or the use of other antioxidants attenuated alcohol-induced liver damage (see Wu and Cederbaum 2003).

### Changes in NADH/NAD^+^ Levels and Gene Activation

NADH and NAD^+^ are involved in many important cellular reactions, and the levels of the two compounds in the cell, as well as their ratio, in many cases determines the rate at which these cellular reactions can proceed. The ratio of NADH to NAD^+^ frequently fluctuates in response to changes in metabolism. Ethanol oxidation, as mentioned before, results in a significant increase in the hepatic NADH/NAD^+^ ratio in both the cytosol and mitochondria (Cunningham et al. 1986; Bailey and Cunningham 1998). In the cytosol, the conversion of ethanol to acetaldehyde by ADH generates NADH, the reducing equivalents of which are then transported into the mitochondria by molecules known as the malate–aspartate shuttle. In the mitochondria, most of the NADH is produced by ALDH. Through both of these pathways, ethanol oxidation vastly increases the availability of NADH to the electron transport chain in the mitochondria.

In addition to its many effects on biochemical reactions, the NADH/ NAD^+^ ratio also may affect the activity (i.e., expression) of certain genes. For example, studies of the effects of reduced food intake (i.e., caloric restriction) on gene expression found that under conditions of caloric restriction, NAD^+^ levels may act as a sensor that regulates the activity of certain genes (Imai et al. 2000). Activation of those genes, in turn, has been shown to extend the lifespan in a wide variety of organisms and to reduce the incidence of age-related diseases, such as diabetes, cancer, immune deficiencies, and cardiovascular disorders (Bordone and Guarente 2005). Changes in the NADH/NAD^+^ ratio resulting from ethanol metabolism might likewise influence gene expression.

## Tissue Damage, Metabolic Derangements, and Disease Associated With Ethanol Metabolism

### Tissue Damage

The direct actions of alcohol (e.g., disordering of membrane components and effects on signaling proteins) and the indirect effects resulting from ethanol metabolism described in the previous sections act in concert to induce tissue damage. In fact, ethanol metabolism often is considered to be the predominant factor causing alcohol-associated tissue damage, particularly through the generation of ROS and oxidative stress in the tissues. ROS are generated during ethanol and acetaldehyde oxidation both by ADH/ALDH and by CYP2E1. The rate of ethanol and acetaldehyde oxidation by ADH and ALDH is determined by the rate with which the NADH generated can pass through the mitochondrial electron transport system. Because the mitochondrial electron transport system requires oxygen and generates ATP, the rate of NADH oxidation depends both on the cell’s oxygen supply and on its demand for ATP. If either of these two factors is limited, electron transport activity is reduced. This has two effects: First, ethanol and acetaldehyde are inefficiently metabolized, and, second, electrons passing through the mitochondrial electron transport chain are “diverted” into forming harmful ROS, mainly superoxide (Hoek et al. 2002). Because ethanol metabolism by ADH and ALDH occurs primarily in the liver, any adverse effects associated with ethanol metabolism by these enzymes and associated ROS production primarily would affect that organ.

In contrast, CYP2E1, which also oxidizes ethanol, particularly following chronic alcohol intake, is found in many tissues in addition to the liver, including the brain, heart, lungs, and certain white blood cells (i.e., neutrophils and macrophages). Accordingly, metabolic consequences of CYP2E1mediated ethanol oxidation would affect numerous tissues. Harmful effects associated with CYP2E1-mediated ethanol metabolism primarily are related to the production of ROS, mainly superoxide and hydroxyl radicals. This ROS production contributes to alcohol-induced damage to a variety of tissues not only by causing oxidative stress but also by enhancing apoptosis triggered by various stimuli. In the liver, CYP2E1mediated ethanol metabolism generates oxidative stress that leads to DNA damage and may thereby play an important role in alcohol-related development of liver cancer (Bradford et al. 2005).

### Effects on Fetal Development

Oxidative stress plays an important role in ethanol-induced damage to the developing fetus (Cohen-Kerem and Koren 2003). Low levels of CYP2E1 are found in prenatal brain (Brezezinki et al. 1999), suggesting that CYP2E1derived ROS could play a role in the development of alcohol-related birth defects, including fetal alcohol syndrome (FAS). Moreover, ROS produced during CYP2E1-mediated ethanol metabolism would likely be particularly harmful because the fetal brain shows only low levels of antioxidant enzyme activity compared with adult brain (Henderson et al. 1999). Researchers have studied whether administration of antioxidants, such as *N*-acetyl cysteine, SAMe, folic acid, and vitamin C, could improve cell survival during fetal ethanol exposure; however, these studies have yielded mixed results.

Nonoxidative metabolism of ethanol by phospholipase D also has been implicated in alcohol-related birth defects. As mentioned earlier, phospholipase D normally is a critical component in cellular signal transduction processes, and the presence of ethanol interferes with these pathways. Alcohol-induced inhibition of these signaling processes during fetal development impairs the proliferation of certain brain cells (i.e., astroglial cells) and may contribute to the reduced brain size (i.e., microencephaly) found in most children diagnosed with FAS (Costa et al. 2004).

### Impairment of Other Metabolic Processes

Chronic ethanol consumption and alcohol metabolism also may influence various other metabolic pathways, thereby contributing to metabolic disorders frequently found in alcoholics, such as fatty liver and excessive levels of lipids in the blood (i.e., hyperlipidemia), accumulation of lactic acid in the body fluids (i.e., lactic acidosis), excessive production of chemical compounds known as ketones in the body (i.e., ketosis), and elevated levels of uric acid in the blood (i.e., hyperuricemia).

The liver is most commonly affected by alcohol-induced damage. The first stage of liver damage following chronic alcohol consumption is the appearance of fatty liver, which is followed by inflammation, apoptosis, fibrosis, and finally cirrhosis. The development of fatty liver is induced by the shift in the redox state of the hepatocytes that results from ethanol metabolism by ADH. This shift in the redox state favors the accumulation of fatty acids, rather than their oxidation. In addition to these metabolic effects, chronic ethanol consumption contributes to the development of fatty liver by influencing the activities of several proteins that help regulate fatty acid synthesis and oxidation (Nagy 2004).

Other metabolic derangements associated with ethanol metabolism result from the fact that ADH and ALDH metabolize not only ethanol but also other compounds. For example, ADH and ALDH oxidize retinol (i.e., vitamin A_1_) to retinal and, subsequently, retinoic acid, which plays an important role in growth and differentiation. In the presence of ethanol, ADH and ALDH may be occupied with ethanol metabolism and retinol metabolism may be inhibited. These interactions may have serious implications for fetal development, stem cell differentiation, maintenance of differentiated tissue function, and the normal structure and function of stellate cells in the liver (Crabb et al. 2001). Chronic alcohol consumption also is associated with disturbances in the metabolism of sulfur-containing amino acids, leading to increased levels of the amino acids glutamate, aspartate, and homocysteine in alcoholic patients. These increases may have serious adverse effects. For example, homocysteine increases and modulates certain nerve signaling processes, particularly during alcohol withdrawal, and increases in homocysteine levels may possibly contribute to the alcoholism-associated tissue shrinkage (i.e., atrophy) observed in brain tissue (Bleich et al. 2004).

### Cancer Risk and Medication Interactions

Chronic alcohol consumption greatly enhances the risk of developing cancer of the esophagus and oral cavity (Seitz et al. 2004) and plays a major role in the development of liver cancer (Stickel et al. 2002). Several mechanisms have been identified that contribute to ethanol-associated tumor development, some of which are related to alcohol metabolism. For example, the acetaldehyde generated during alcohol metabolism promotes cancer development, as does induction of CYP2E1 leading to ROS formation (Pöschl and Seitz 2004).

Induction of CYP2E1 following heavy alcohol consumption also has other potentially harmful consequences:

- Enhanced alcohol metabolism by CYP2E1 contributes to alcoholics’ metabolic tolerance for ethanol, thereby promoting further alcohol consumption.
- CYP2E1 metabolizes not only ethanol but also numerous other compounds, including many medications. Induction of CYP2E1 also affects the metabolism of these medications, potentially making them less effective. Medications metabolized by CYP2E1 include, among others, propranolol (which is prescribed for the treatment of hypertension and disturbances of the heart rhythm), the pain medication acetaminophen, the blood thinner warfarin, and the sedative diazepam.
- CYP2E1 activates numerous pro-carcinogens found in tobacco smoke. Concurrent drinking and smoking significantly increase the risk of esophageal and laryngeal cancer.

## Concluding Remarks

Today, much is known about ethanol metabolism, particularly its oxidation by ADH and ALDH in the liver. However, gaps remain in researchers’ understanding of certain aspects of ethanol metabolism. For instance, the relative contributions of ADH, catalase, and microsomal enzymes to ethanol metabolism in the brain have not yet been determined. Other questions include, How do oxidative pathways via catalase relate to fatty acid oxidation through mitochondrial pathways? Could ethanol metabolism affect satiety, insulin resistance, liver function, and fat formation by indirectly modulating the expression of certain genes, similar to the processes observed under conditions of caloric restriction? Can the fluctuations in cellular redox state resulting from ethanol metabolism directly influence ethanol-induced liver injury? What are the mechanisms responsible for the hypersensitivity of ethanol-exposed hepatocytes in TNF-α– induced injury? What is the role of changes in redox state on the expression of cytokines that promote or prevent inflammatory reactions? What are the interactions between ethanol metabolism, diabetes, and obesity? Answers to these and other questions will further elucidate the mechanisms underlying ethanol’s metabolism and their regulation, as well as the effects that alcohol metabolism and its byproducts have on all tissues and organs throughout the body. In addition, a deeper understanding of these processes will allow researchers to design intervention strategies that may ameliorate the harmful effects of alcohol and its metabolites.

## Figures and Tables

**Table 1:** Human Alcohol Dehydrogenase (ADH) Isozymes

Class	Gene Nomenclature		Protein	*K*_m_ mM	V_max_ min^−1^	Tissue
	New	Former				
I	*ADH1A*	*ADH1*	α	4.0	30	Liver
	ADH1B*1	ADH2*1	β_1_	0.05	4	Liver, Lung
	ADH1B*2	ADH2*2	β_2_	0.9	350	
	ADH1B*3	ADH2*3	β_3_	40.0	300	
	ADH1C*1	ADH3*1	γ_1_	1.0	90	Liver, Stomach
	ADH1C*2	ADH3*2	γ_2_	0.6	40	
II	*ADH4*	*ADH4*	π	30.0	20	Liver, Cornea
III	*ADH5*	*ADH5*	χ	>1,000	100	Most Tissues
IV	*ADH7*	*ADH7*	σ(μ)	30.0	1,800	Stomach
V	*ADH6*	*ADH6*		?	?	Liver, Stomach

NOTE: The *ADH1B* and *ADH1C* genes have several variants with differing levels of enzymatic activity. *K*_m_ is a measurement used to describe the activity of an enzyme. It describes the concentration of the substance upon which an enzyme acts that permits half the maximal rate of reaction. It is expressed in units of concentration. V_max_ is a measure of how fast an enzyme can act. It is expressed in units of product formed per time.

**Table 2:** Ethanol Metabolites and Adducts Generated During Ethanol Metabolism

Metabolites and Adducts	Source
Acetaldehyde	Ethanol metabolism
Malondialdehyde (MDA)	Nonenzymatic lipid peroxidation of unsaturated fatty acids, breakdown of arachidonic acid in platelets
4-hydroxynonenal (HNE)	Lipid peroxidation of long-chain polyunsaturated fatty acids
Malondialdehyde-Acetaldehyde Adduct (MAA)	Hybrid adducts with malondialdehyde and acetaldehyde
Hydroxyethyl radical (HER)	Ethanol oxidation in the presence of iron

## Footnotes

1 A millimole represents a concentration of 1/1,000 (one thousandth) molecular weight per liter (mol/L).

2 *K*_m_ is a measurement used to describe the activity of an enzyme. It describes the concentration of the substance upon which an enzyme acts that permits half the maximal rate of reaction.

3 Biogenic amines are organic compounds formed during biochemical processes in plants and animals that carry a nitrogen atom as a central molecule.
